# Substrate-induced strain in 2D layered GaSe materials grown by molecular beam epitaxy

**DOI:** 10.1038/s41598-020-69946-4

**Published:** 2020-07-31

**Authors:** Cheng-Wei Liu, Jin-Ji Dai, Ssu-Kuan Wu, Nhu-Quynh Diep, Sa-Hoang Huynh, Thi-Thu Mai, Hua-Chiang Wen, Chi-Tsu Yuan, Wu-Ching Chou, Ji-Lin Shen, Huy-Hoang Luc

**Affiliations:** 1 Department of Electrophysics, National Chiao Tung University, Hsinchu, 30010 Taiwan; 2 Department of Physics, Chung Yuan Christian University, Chung Li, 32056 Taiwan; 3 Faculty of Physics, Hanoi National University of Education, Cau Giay, Hanoi, Vietnam

**Keywords:** Materials science, Nanoscience and technology

## Abstract

Two-dimensional (2D) layered GaSe films were grown on GaAs (001), GaN/Sapphire, and Mica substrates by molecular beam epitaxy (MBE). The in situ reflective high-energy electron diffraction monitoring reveals randomly in-plane orientations of nucleated GaSe layers grown on hexagonal GaN/Sapphire and Mica substrates, whereas single-orientation GaSe domain is predominant in the GaSe/GaAs (001) sample. Strong red-shifts in the frequency of in-plane ${E}_{2g}^{2}$ vibration modes and bound exciton emissions observed from Raman scattering and photoluminescence spectra in all samples are attributed to the unintentionally biaxial in-plane tensile strains, induced by the dissimilarity of symmetrical surface structure between the 2D-GaSe layers and the substrates during the epitaxial growth. The results in this study provide an important understanding of the MBE-growth process of 2D-GaSe on 2D/3D hybrid-heterostructures and pave the way in strain engineering and optical manipulation of 2D layered GaSe materials for novel optoelectronic integrated technologies.

Since the first discovery of atomically single-layer graphene along with its exotic physical phenomena and unique applications, fresh blood of two-dimensional (2D) materials family including hexagonal (hex) boron-nitride, transition metal dichalcogenide (TMDs), group III-metal chalcogenides, and so on have ignited more than a decade intensive attention^1–6^. Those novel 2D materials with an adjustable wide-range bandgap from 0.4 to 6.0 eV not only overcome the gapless-band obstacle of graphene^7^ also exhibit advanced integrations of exceptional properties including anisotropic electrical and optical behaviors, high charge density of states, quantum confinement effects, and flexibility. Those merits along with a variety of their hybrid-heterostructures make 2D layered materials fascinating to fundamentally physical researches and to high-end applications in electronics, optoelectronics, photonics, and flexible devices^8–12^. Among 2D materials, GaSe, a typical member of group III-metal chalcogenide, is now attracting much attention due to its thickness-dependent opto-electronic properties that showing an opposite trend in other TMDs^13–15^. It also exhibits high on/off current ratio^16^, excellent photoresponses^14,15^, anisotropic Hall-mobility, and high resistivity which allow very low dark current in photodetectors^17^^,^ and superior second-harmonic generation^3,18^. More attractively, *ex-situ* strain engineering applied into the 2D layered materials is a powerful approach to dynamically modify the electronic band structure as well as strengthen other optical properties, considering for the new generation of flexible optoelectronic devices. The number of techniques has been proposed recently to manipulate mechanical deformation, such as bending of the flexible substrate^19–21^, controlling of the wrinkled structure^22,23^, elongating of the substrate^24^, stretching of the piezoelectric substrate^25^, suspended 2D layers on a hole-patterned substrate^26^. For GaSe, the strong shifting of the conduction band minimum of 2D-GaSe layers under ex situ strain engineering techniques have been confirmed theoretically^23,27^ as well as experimentally^19,20^, resulting in a reduction of bandgap energy with increasing the elastic strain degree. In another aspect, it seems reasonable to assume that the van der Waals (vdW) interaction allows 2D layered materials, GaSe, in particular, can be readily grown on different kinds of substrates regardless of the lattice mismatch^28,29^. However, there still have inconsistencies in the structural and electronic characteristics of the 2D epitaxial GaSe layers, which normally stem from the unintentional strain generation, the charge transfer and hence the interface dipole formation between layer and substrate during the MBE growth^30–32^. Unfortunately, there is no literature making a comprehensive understanding of the internal strain-induced physical properties in 2D GaSe epitaxial layers grown on various substrates. Thus, from this point of view along with the aim of exploring a possible in situ strain engineering approach, we grew the GaSe epitaxial layers on various substrates including GaAs (001), Mica, and GaN/Sapphire platform under an identical growth condition by molecular beam epitaxy (MBE). The main reason to choose these substrates is to address three distinguished layer/substrate surface configurations for comparison (based on two factors: (1) symmetric surface structure and (2) lattice mismatch), which cover a wide range of 2D GaSe-based hybrid-heterostructures from hex-2D/cubic-3D (GaSe/GaAs (001)) and hex-2D/hex-3D (GaSe/GaN/Sapphire) to hex-2D/hex-2D (GaSe/Mica). According to the results, we propose that the symmetric similarity between the GaSe layer and substrate surface plays an important role in the strain generation inside the layer during the epitaxial growth and other optical properties of the materials rather than the lattice mismatch. Besides that, the important information of MBE process and crystal properties of these hybrid-heterostructures explored from this study would help to pave the way for further investigations on electronic behaviors at the layer/substrate interface, including the charge transfer, inter-diffusion, interface dipole, electronic band dispersion characteristics, band bending in the heterojunctions, and so on^33,34^. These are crucial experiments in order to inherit advantages of the commercial 3D-semiconductor substrates and exceptional properties of 2D-GaSe materials and discover novel hybrid-heterostructures for electronic and optoelectronic device applications in the future.

## Results and discussion

**Figure 1:**
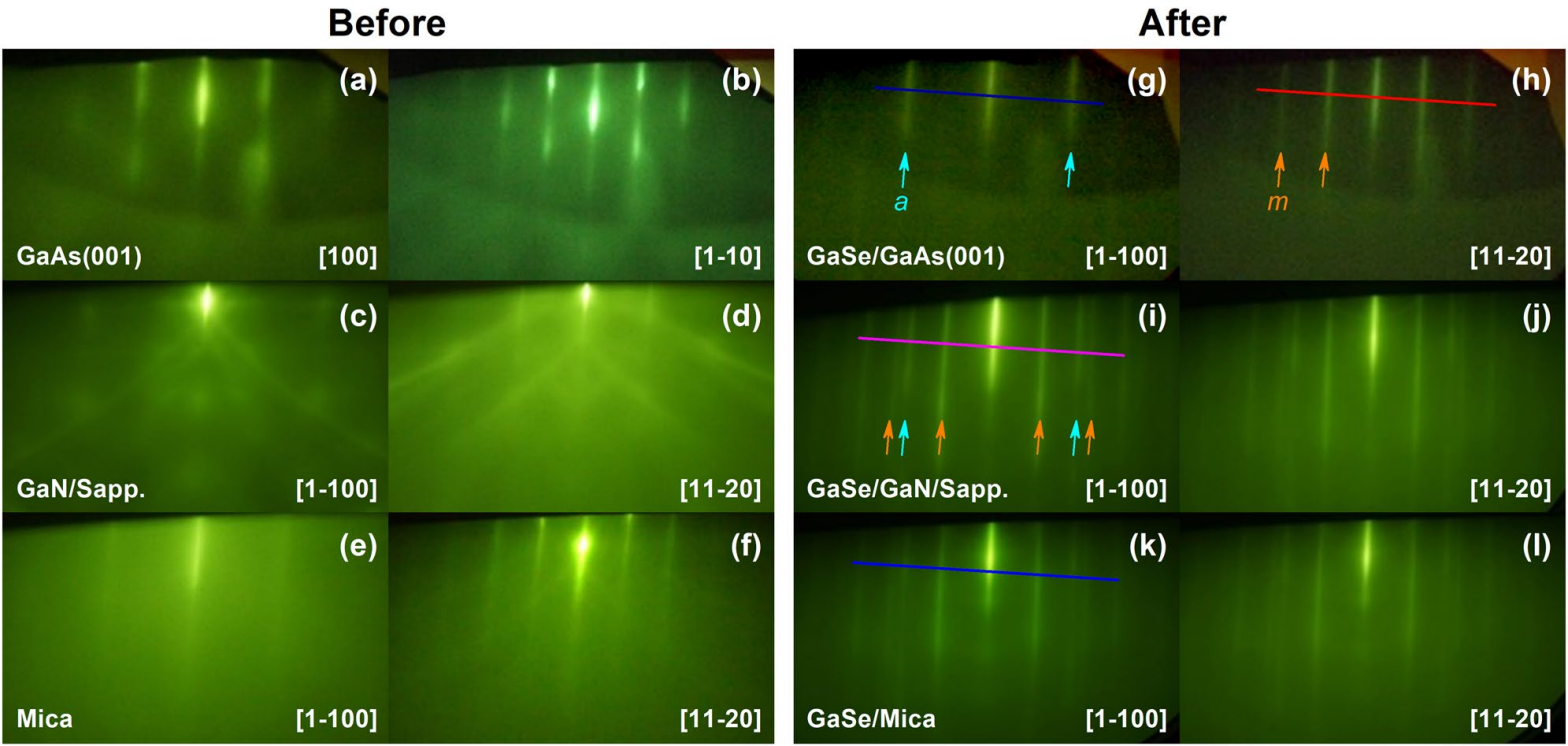
RHEED patterns monitored before (**a**)–(**f**) and after (**g**)–(**l**) the GaSe deposited on various substrates.

Figure 1 shows the in situ reflective high-energy electron diffraction (RHEED) patterns of all samples before (Fig. 1a-f) and after (Fig. 1g-l) the growth of GaSe layers on various substrates. For the growth on GaAs (001) substrate, the RHEED pattern observed along [100] substrate surface direction (Fig. 1a) turned quickly into the sharp-streaky patterns after a few minutes depositing GaSe layer (Fig. 1g), which was reappeared after every 60° azimuthal substrate rotation and then preserved until the end of the growth. By 30° azimuthally sample rotating from Fig. 1g, the streaky-pattern with shorter spacing was observed as shown in Fig. 1h. Besides, the spacing ratio between streaks in Fig. 1g and in Fig. 1h was close to $\sqrt{3}$. These indicate that the six-fold symmetry GaSe crystalline layer had been deposited on the GaAs (001) substrate, where the $[1\bar{1}00]$ direction of GaSe hexagonal surface structure was aligned to the [100] direction of cubic GaAs surface.^35^.

**Figure 2:**
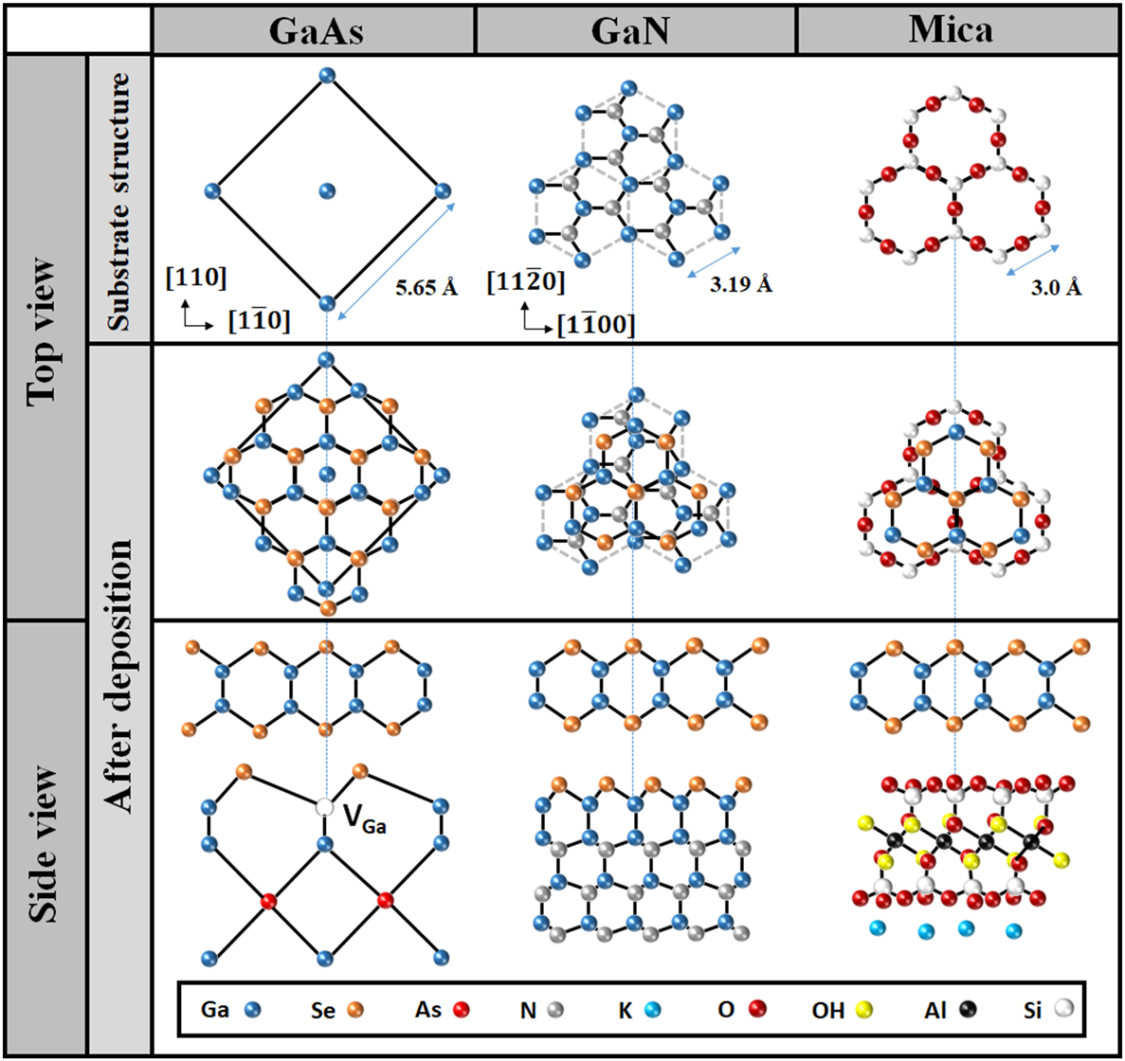
Illustration of perspective atomic-stacking configuration between GaSe layers and substrates.

**Figure 3:**
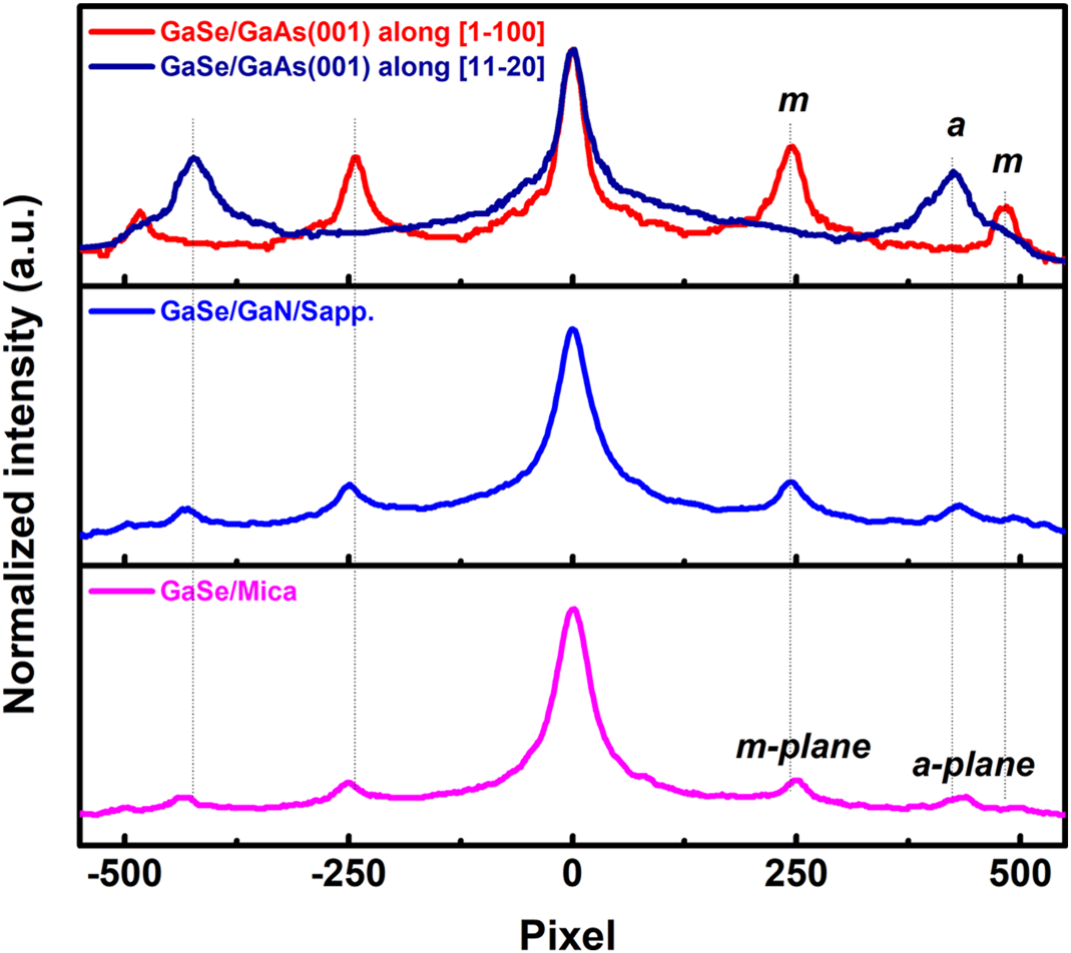
RHEED intensity profiles of the GaSe layers on various substrates.

The long-streaky RHEED patterns were also observed during the GaSe depositing on GaN/Sapphire platform and Mica substrate as can be seen in Fig. 1i,j and Fig. 1k,l, respectively. Interestingly, these RHEED patterns exhibited the co-existence of *a*-plane and *m*-plane streaks of the hexagonal GaSe structure on either the $[1\bar{1}00]$ or $[11\bar{2}0]$ observed directions, attributed to the randomly in-plane orientation of the nucleated GaSe flakes^36^. This was contrary to the GaSe/GaAs (001) RHEED patterns, where the *a*-plane and *m*-plane streaks were identified separately from $[1\bar{1}00]$ and $[11\bar{2}0]$ observed directions (Fig. 1g and 1h), respectively. Side and top perspective stacking orientations of the GaSe layers deposited on various substrates are illustrated in Fig. 2. One can be noticed that the hexagonal basal plane of GaSe is symmetrically equivalent to that of GaN or Mica substrate, while it is very distinct from the cubic basal plane of GaAs substrate. Thus, it could lead to the statement that the uniformity of the in-plane orientation of the GaSe layer would prefer to grow on a symmetrically non-equivalent substrate surface. The coexistence of two domain orientation of the GaSe flakes grown on hexagonal-surface substrates by MBE was usually observed, but its origin has been clearly understood^31,36–38^. This may relate to the local surface energy fluctuations or the kinetics of the MBE growth process^31^. Herein, a possible explanation is that the in-plane lattice orientation of the nucleated GaSe flakes could be governed by the potential surface energy distribution of the substrate, where the GaSe seeds would be deposited and aligned to the substrate orientation that has more energetically favorable. The non-uniform orientation of the GaSe layers observed in GaSe/GaN/Sapphire and GaSe/Mica could come from the small difference in the favorable surface energy between $[1\bar{1}00]$ and $[11\bar{2}0]$ substrate direction; thus, at the initial growth stage, the nucleated GaSe flakes have a higher possibility to be oriented randomly on both directions. On the other hand, the favorable energy between [100] and $[1\bar{1}0]$ GaAs surface direction may be so different that the nucleated GaSe flakes were preferentially deposited along the more energetically favorable direction, introducing a high-ordered orientation of the GaSe layer. The streak spacing profiles (Fig. 3) extracted from the straight-lines in Fig. 1g–l revealed that the in-plane surface lattice constant of the GaSe layer on GaAs (001) was larger than that of those layers grown on GaN/Sapphire and Mica substrates. These lattice constants are calculated from the streak spacing to be 3.91 Å, 3.85 Å, and 3.78 Å for the growth on GaAs (001), GaN/Sapphire, and Mica, respectively. The calculation is based on the translation between the streak spacing in the reciprocal lattice and the number of pixels achieved from the RHEED images^39^ (see Table S1). All values are slightly longer than the theoretical in-plane lattice constant of GaSe bulk of 3.755 Å. It means that all GaSe layers demonstrated a small in-plane tensile surface strain, where the GaSe/GaAs (001) sample had the highest degree of the strain among these samples.

**Figure 4:**
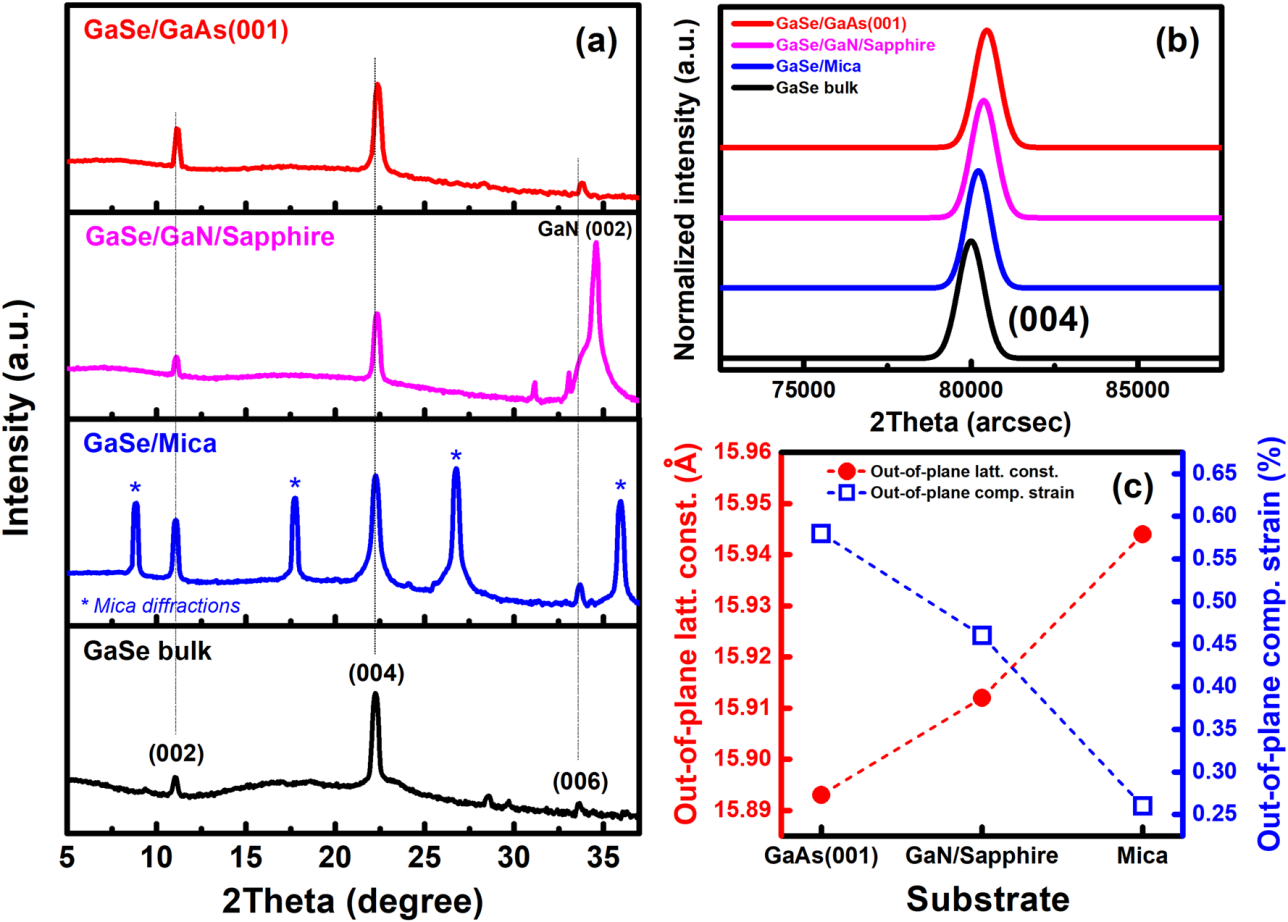
Long-range (**a**) and near (004) peak (**b**) XRD 2θ-scans of GaSe bulk and GaSe films grown on various substrates. (**c**) Out-of plane lattice constants and compressive strains of GaSe layers.

To give further information about the lattice parameters of the GaSe layers, high-resolution X-ray diffraction (XRD) 2θ-scans of all samples as well as of the GaSe bulk were addressed carefully with a scan step of 0.01°. As can be seen in Fig. 4a, (002) and (004) characteristic XRD planes of the GaSe layers are easy to observe in all samples, located at 2θ angles of ~ 11.1° and ~ 22.2°, respectively. In comparison to GaSe bulk, all (004) diffraction positions of three samples shifted to higher 2θ angles (Fig. 4b), indicating the presence of out-of-plane compressive strains in these GaSe layers. The theoretical in-plane lattice constants of GaAs (001), GaN, and Mica are 5.65 Å, 3.19 Å, and 5.2 Å, introducing the in-plane lattice mismatches of − 33.6%, + 17.6%, and − 27.9%, respectively (“*minus*” indicates to a tensile in-plane mismatch and vice versa). One may expect that these huge in-plane lattice mismatches may contribute to the degree of out-of-plane strain in the layers; however, there is no evidence to describe this relationship in our presented data. Obviously, the epitaxial stacking between GaSe intra-layers in these samples was governed by van der Waals interaction regardless of either sign or magnitude of their in-plane lattice mismatches^28^. In addition, the extracted out-of-plane lattice strains from the (004) diffraction peaks of the GaSe layers grown on GaAs (001), GaN/Sapphire, and Mica substrate (Fig. 4c) show that the 2D-hex GaSe/3D-cubic GaAs (001) sample has suffered a largest out-of-plane compressive strain of 0.58%, while the smallest value of 0.26% belongs to the 2D-GaSe/2D-Mica sample (Table 1). It suggests that the equivalent symmetry of the basal plane between the layer and substrate may play a role in this out-of-plane strain variation.

**Table 1:** Extracted lattice parameters and optical properties of the GaSe layers grown on different substrates and the GaSe bulk.

Substrate	In-plane surface lattice constant (Å)	Out-of plane lattice constant (Å)	Out-of plane compressive strain (%)	E^2^_2g_ mode (cm^−1^)	Biaxial in-plane tensile strain (%)	PL emission (eV)
GaAs (001)	3.909	15.89	0.58	204.2	0.84	1.745
GaN/Sapp	3.849	15.91	0.46	205.2	0.70	1.840
Mica	3.780	15.94	0.26	205.7	0.63	1.902
GaSe bulk	3.755	15.98	0	210.2	0	2.045

**Figure 5:**
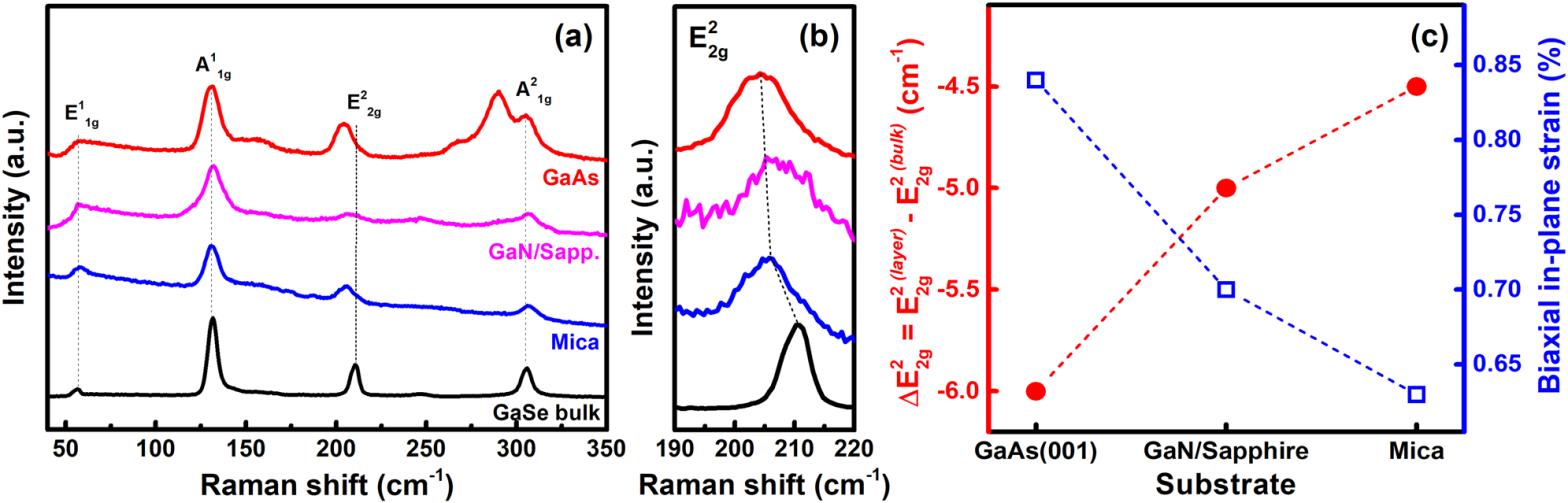
(**a**) Raman scattering spectra of GaSe bulk and GaSe films grown on various substrates, (**b**) An enlarged Raman spectra near E^2^_2g_ peaks, and (**c**) Biaxial in-plane lattice strain of these GaSe layers extracted from the red-shift of ${E}_{2g}^{2}$ Raman active mode.

To explore more deeply into structural and optical properties of the samples, Raman scattering and photoluminescence (PL) spectra were carried out. As observed in Fig. 5a, four typical Raman active modes of the GaSe layers were clearly identified in all samples, including ${E}_{1g}^{1}$ (~ 56 cm^−1^), ${A}_{1g}^{1}$ (~ 131 cm^−1^), ${E}_{2g}^{2}$ (~ 205–210 cm^−1^), and ${A}_{1g}^{2}$ (306 cm^−1^) mode. Interestingly, the in-plane vibration ${E}_{2g}^{2}$ mode of the GaSe epitaxial layers exhibited strong red-shifts (Fig. 5b) as compared to that of GaSe bulk (210.2 cm^−1^), where the ${E}_{2g}^{2}$ mode located at 204.2 cm^−1^ (on GaAs), 205.2 cm^−1^ (on GaN/Sapphire), and 205.7 cm^−1^ (on Mica). This is in contrast to the likely non-shifting of other out-of-plane vibration modes and as a result of the in-plane tensile strains in our GaSe films. Detail information including the position and full-width at half-maximum (FWHM) of each Raman peak of the samples in comparison to other reports is shown in Table S2 in Supplementary Information. Basically, the strain induced by mechanical deformation could be categorized into four kinds: biaxial, armchair, zigzag, and shear strain. In the case of the unintentional strain generation during the 2D layered material growth, we assume that only biaxial strain contributes to the 2D GaSe strained-layers^40^. The strain-induced Raman frequency shift $\partial {\omega }_{m}$ at the frequency of the *m* Raman active mode ${\omega }_{m}^{0}$ under a biaxial strain (*ε*_*bxy*_) could be derived through the mode-Grüineisen parameter ${\gamma }_{m}$ as:^40–42^

1 $${\gamma }_{m}=-\frac{1}{2{\omega }_{m}^{0}}\frac{\partial {\omega }_{m}}{{\partial \varepsilon }_{bxy}}$$

Because of the crystal symmetry retaining under biaxial strain, resulting in no degeneracy lift, thus the Eq. (1) can be rewritten as:

2 $${\varepsilon }_{bxy}=-\frac{\Delta {\omega }_{m}}{2{\gamma }_{m}{\omega }_{m}^{0}}$$

By referring the ${E}_{2g}^{2}$ GaSe mode-Grüineisen parameter of 1.7^41^^,^ the calculated biaxial strain in the GaSe layers grown on GaAs (001), GaN/Sapphire, and Mica substrate are 0.84%, 0.7%, and 0.63%, respectively (Fig. 5c). On the other hand, the non-shifting behavior of ${E}_{1g}^{1}$ (~ 56 cm^−1^) in-plane vibration mode is well consistent with another report, where the Ga-Ga bond length is hard to vary in case of isotropic expansion initiated by a small biaxial strain (Fig. S1)^40^. Thus, this result reinforces our assumption about the existence of only biaxial strain in the samples. Moreover, the variation of biaxial in-plane strain in our samples grown on various substrates is in agreement with the surface symmetric equivalency between layer and substrate instead of the lattice-mismatched values. The highest surface symmetric equivalency, GaSe/Mica sample, would introduce the lowest biaxial strain of 0.63%, for example. Consequently, we propose that the degree of the induced-strain during the materials growth is mainly affected by the structure of the substrate surface. Herein, the biaxial in-plane tensile strain variation could correlate with the out-of-plane compressive strain (Fig. 4c; however, this relationship is hard to define quantitatively in 2D-vdW crystals, especially with a relatively small magnitude of the substrate-induced strains in our case.

**Figure 6:**
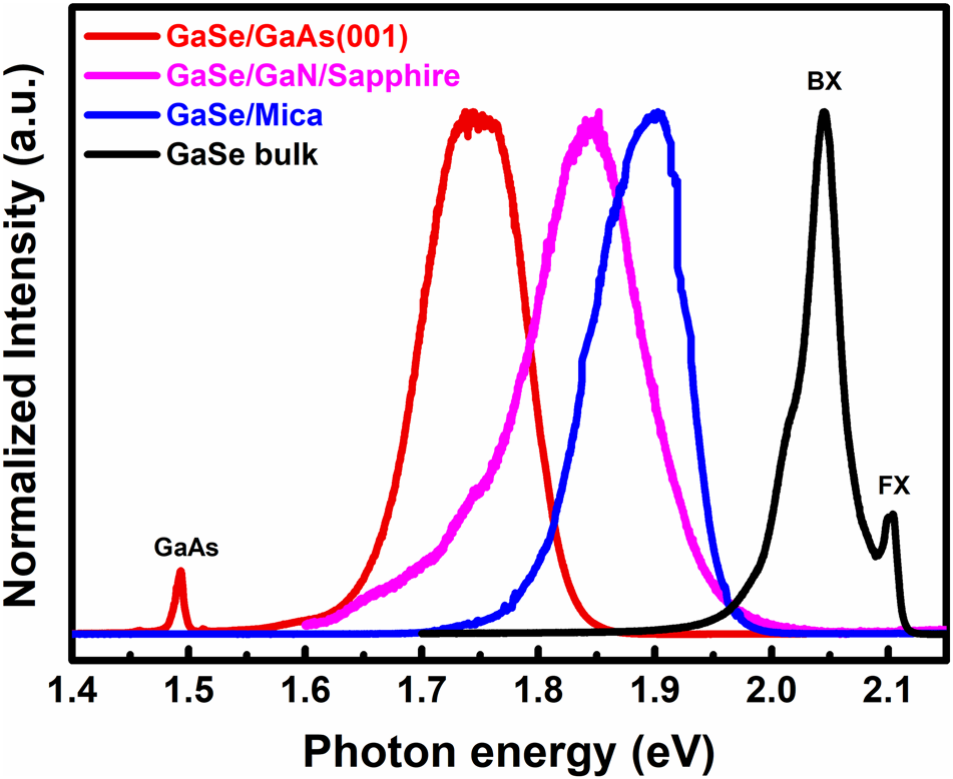
PL spectra of the GaSe epilayers on various substrates in comparison to GaSe bulk measured at 10 K.

The PL spectra of GaSe bulk and GaSe thin films grown on GaAs, GaN/Sapphire, and Mica were studied at 10 K as shown in Fig. 6. For GaSe bulk, there are two main features, including the free exciton emission (FX) (sharp-narrow peak at 2.104 eV) and the bound exciton emission (BX) (the lower energy peak with phonon-replica like shoulders near ~ 2.0 eV)^43^. As compared to GaSe bulk, the PL emissions of all GaSe films exhibited broadened, non-separated FX features, and a strong shift to lower energies, locating at 1.745, 1.84, and 1.90 eV for the growth on GaAs, GaN/Sapphire, and Mica substrate, respectively. The broadened-PL peaks and the non-separated FX emissions in these GaSe thin films (fitted-PL peaks are shown in Figure S2 and Table S3) are completely understandable when comparing to bulk materials. As a result, the broadened PL peaks of the GaSe thin films have resembled as the BX emission of GaSe bulk^35^. It is easy to notice that the tendency of the PL peak variations is in good agreement with that of in-plane tensile strain as extracted above from the Raman spectra of our samples as well as other reports. Remarkably, the results demonstrate that the bandgap of the GaSe epitaxial layer is easily tunable on a large range from 1.74 to 1.9 eV by substrate replacing from GaAs (001) to Mica, depending on each particular optoelectronic application.

In conclusion, the 2D GaSe layers grown by MBE on GaAs (001), GaN/Sapphire, and Mica substrate have been demonstrated in this study. The in situ RHEED monitoring reveals that the growth of 2D-hex GaSe/3D-cubic GaAs (001) introduces a uniform in-plane orientation in the GaSe layer, while the random GaSe orientations grown on GaN/Sapphire and Mica substrates were observed. The lattice parameters, as well as the strain behavior in these samples, were figured out comprehensively from RHEED, XRD, and Raman scattering measurements. It is pointed out that the GaSe layers have experienced a biaxial in-plane tensile strain during the epitaxial growth, mainly governed by the symmetric discrepancy in surface structure between the GaSe layer and the substrate. Interestingly, this contributes to a strong variation in the bandgap of the GaSe epitaxial layers from 1.74 to 1.9 eV. The results presented in this study provide an understanding of the influence of substrate surfaces on the lattice dynamics of the 2D-hexagonal GaSe layers during the MBE growth.

## Methods

2D layered GaSe thin films were grown by SVTA MBE system operating at a background pressure of 1.0 × 10^–10^ torr using the standard high purity sources of (7 N)-Ga and (6 N)-Se element. Firstly, all substrates including GaAs (001), commercial muscovite mica, and GaN/Sapphire grown by metalorganic vapor deposition (MOCVD) were cleaned by acetone, followed by rinsing in DI water and drying with nitrogen before loading into the MBE chamber. For the GaSe/GaAs (001) growth, the substrate was first heated up to 600 °C under an ultra-high vacuum of ~ 7.0 × 10^–9^ torr and kept in a certain period to assure removing completely the surface native oxides, then ramping down the substrate temperature to the growth temperature (T_g_) of 425 °C. For the growth of GaSe on both Mica and GaN/Sapphire, the substrates were directly heated up to 425 °C without any pre-annealing processes. The growth parameters of all samples were kept the same, where the Ga source shutter was released in 2 min prior to opening the Se shutter with the beam equivalent pressures of 1.4 × 10^–7^ torr for Ga and 7.8 × 10^–7^ torr for Se, resulting in a Se/Ga flux ratio of ~ 5.6. The growth time of all samples was four hours, approximately a layer thickness of ~ 300 nm. All samples were cooled down at the same cooling rate before loading out the chamber. This is to minimize the possible difference in the thermal expansion mismatch-induced strain between the samples during the cooling process. The surface configuration during the material growths was carefully recorded by the in situ RHEED monitoring. The structural and optical properties of all samples were comprehensively characterized and analyzed by XRD 2θ-scans using SRA M18XHF diffractometer with Cu-K_α_ radiation (*λ* = 1.54056 Å), micro-Raman scattering, and 10K-PL spectra using LabRam iHR550 HORIBA spectrometer at the laser excitation wavelengths of 488 nm (Ar^+^), and 325 nm (He–Cd), respectively.

## Supplementary information

Supplementary Information.
