# Supplementary material for: Substrate-induced strain in 2D layered GaSe materials grown by molecular beam epitaxy
Source: Sci Rep. 2020 Jul 31;10:12972. doi: 10.1038/s41598-020-69946-4 (PMC7395717; doi:10.1038/s41598-020-69946-4)
Supplement: Supplementary file 1 — Supplementary Information. [file 41598_2020_69946_MOESM1_ESM.docx]

**SUPPLEMENTARY INFORMATION for**

**Substrate-induced strain in 2D layered GaSe materials grown by molecular beam epitaxy**

Cheng-Wei Liu^1^, Jin-Ji Dai^1^, Ssu-Kuan Wu^1^, Nhu-Quynh Diep^1^, Sa-Hoang Huynh^1^, Thi-Thu Mai^1^, Hua-Chiang Wen^1^, Chi-Tsu Yuan^2^, Wu-Ching Chou^1*^, Ji-Lin Shen^2^, and Huy-Hoang Luc^3^

*^1^Department of Electrophysics, National Chiao Tung University, Hsinchu 30010, Taiwan.*

*^2^Department of Physics, Chung Yuan Christian University, Chung Li 32056, Taiwan.*

*^3^Faculty of Physics, Hanoi National University of Education, Cau Giay, Hanoi, Vietnam.*

^*^*Correspondence and requests should be addressed to Prof. W.C. Chou*

*Email: wuchingchou@mail.nctu.edu.tw*

**Table S1.** Measured streak spacing and calculated average in-plane surface lattice constant of GaSe layers on various substrates

| **Samples** | **Number of pixels of a-/m-plane spacing** | **Streak spacing between a/m-plane (Å^-1^)** | **Streak spacing ratio (a/m)** | **Average in-plane surface lattice constant (Å)** |
| --- | --- | --- | --- | --- |
| **GaSe/GaAs(001)** | 424/243 | 3.227/1.849 | 1.745 | **3.909** |
| **GaSe/GaN/Sapp.** | 432/246 | 3.288/1.872 | 1.756 | **3.849** |
| **GaSe/Mica** | 439/251 | 3.341/1.910 | 1.749 | **3.780** |

**Table S2:** Extracted position and FWHM of three Raman active-modes of the GaSe samples

| **Sample** | **A^1^_1g_ Pos./FWHM (cm^-1^)** | **E^2^_2g_ Pos./FWHM (cm^-1^)** | **A^2^_1g_ Pos./FWHM (cm^-1^)** | **Thickness** | **Ref.** |
| --- | --- | --- | --- | --- | --- |
| **GaSe bulk** | 131.5/5.8 | 210.2/5.3 | 306.2/6.7 | N/A | This work |
| **GaSe/GaAs (001)** | 131.5/10.4 | 204.2/11.2 | 306.3/15.0 | 300 nm |  |
| **GaSe/GaN/Sapp.** | 131.6/13.1 | 205.2/15.0 | 306.3/13.3 |  |  |
| **GaSe/Mica** | 131.6/8.9 | 205.7/9.7 | 306.2/10.7 |  |  |
| **GaSe/GaAs (001)** | 132.0/~8 | N/A | N/A | 200 nm | Acta Phys. Pol. A 136, 4 (2019) |
| **GaSe/GaN/Sapp.** | 132.0/~9 | N/A | N/A | 75 nm | J. Appl. Phys. 121, 094302 (2017) |
| **GaSe/Mica** | 131.7/~9 | N/A | N/A | 30 layers | Nano Lett. 15, 5, 3571–3577 (2015) |


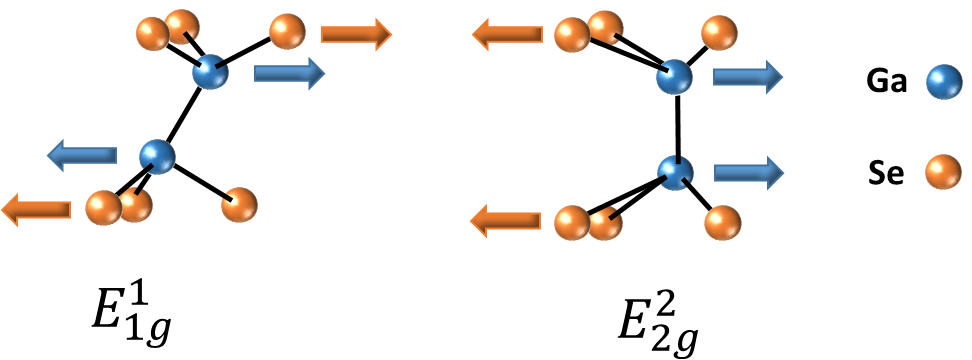


**Figure S1.** Schematic illustration of in-plane vibration $E_{1g}^{1}$ and $E_{2g}^{2}$ modes of 2D-GaSe materials


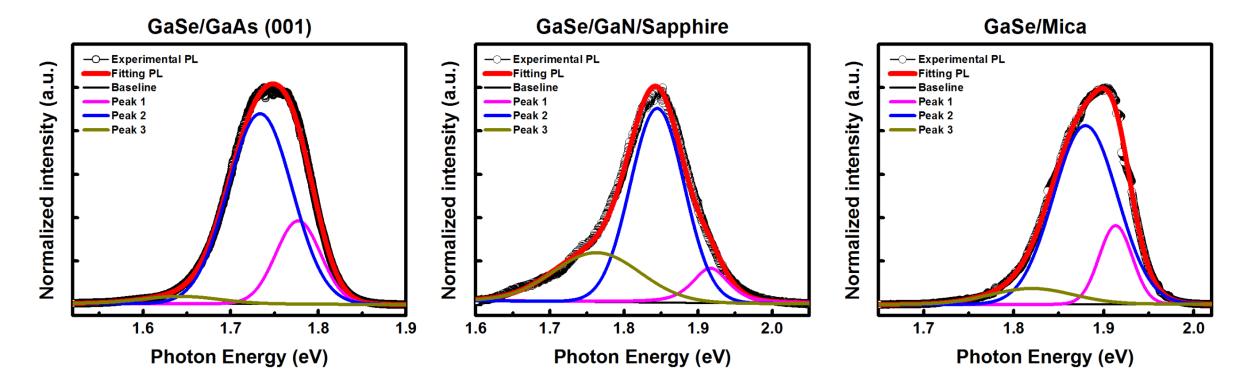


**Figure S2:** Fitting PL emissions of as-grown GaSe films. Peaks 1, 2, and 3 could be considered as free-exciton, bound-exciton, and defect level emission, respectively [C. Wei *et.al.*, ***Sci. Rep. 6***, (2016)].

**Table S3:** Fitting parameters of 10K-PL emission of as-grown GaSe films

| **Sample** | **Thickness (nm)** | **Free-exciton** | | **Bound-exciton** | | **Ref.** |
| --- | --- | --- | --- | --- | --- | --- |
|  |  | Peak (eV) | FWHM (meV) | Peak (eV) | FWHM (meV) |  |
| **GaSe bulk** | N/A | 2.103 | 12 | 2.046 | 30 | This work |
| **GaSe/GaAs** | 300 | 1.777 | 60 | 1.737 | 86 |  |
| **GaSe/GaN/Sapp.** | 300 | 1.915 | 58 | 1.842 | 86 |  |
| **GaSe/Mica** | 300 | 1.920 | 43 | 1.880 | 84 |  |
| **GaSe bulk** (measured at 60K) | 10µm | 2.102 | 10 | 2.086 | 70 | C. Wei *et.al.*, ***Sci. Rep. 6***, (2016) |

Based on the PL-fitting data, the quality of our bulk GaSe is comparable with that of bulk GaSe in the literature, where their free-exciton emissions located at ~2.1 eV. On the other hand, the FWHMs of bound-exciton emissions of the as-grown samples (~86 meV) were ~3 times larger than that of bulk GaSe. This reveals a lower quality of these epitaxial layers, and agrees well with Raman results.
